# Hypertensive retinopathy and its association with cardiovascular, renal and cerebrovascular morbidity in Congolese patients

**DOI:** 10.5830/CVJA-2014-045

**Published:** 2014

**Authors:** Nelly N Kabedi, David L Kayembe, Jean-Claude Mwanza, François B Lepira, Tharcisse K Kayembe

**Affiliations:** Department of Ophthalmology, School of Medicine, University of Kinshasa, Kinshasa, Democratic Republic of Congo; Department of Ophthalmology, School of Medicine, University of Kinshasa, Kinshasa, Democratic Republic of Congo; Department of Ophthalmology, School of Medicine, University of North Carolina at Chapel Hill, Chapel Hill, North Carolina, USA; Division of Nephrology, School of Medicine, Department of Internal Medicine, University of Kinshasa, Kinshasa, Democratic Republic of Congo; Department of Neurology, School of Medicine, University of Kinshasa, Kinshasa, Democratic Republic of Congo

**Keywords:** hypertension, hypertensive retinopathy, left ventricular hypertrophy, chronic kidney disease, stroke

## Abstract

**Background:**

Signs indicating hypertensive retinopathy can help determine the extent of hypertensive cardiovascular, renal and cerebrovascular damage.

**Objectives:**

To study the association between hypertensive retinopathy and cardiovascular, renal and cerebrovascular changes, and to determine the predictors of hypertensive retinopathy in Congolese patients.

**Methods:**

A total of 159 hypertensive subjects (mean age: 58.9 ± 13.2 years) were enrolled from the cardiology out-patient clinic. Retinopathy grade was assessed on direct ophthalmoscopy. Hypertensive cardiovascular, renal and cerebrovascular changes were indicated by left ventricular hypertrophy (LVH), chronic kidney disease (CKD) and stroke, respectively.

**Results:**

Hypertensive retinopathy was present in 83.6% of the patients (grade 1: 42.1%; grade 2: 11.3%; grade 3: 23.3%; grade 4: 6.9%). There was no association between hypertensive retinopathy and the presence or absence of LVH (86.5 vs 73.3%, χ^2^ = 1.53, *p* = 0.21), chronic kidney disease (89.3 vs 83.3%, χ^2^ = 0.12, *p* = 0.73) or stroke (85.7 vs 83.2%, χ^2^ > 0.001, *p* = 0.99). On multivariate logistic regression, CKD was the most significant predictor of severe hypertensive retinopathy, with an odds ratio of 4.4.

**Conclusion:**

No association was found between hypertensive retinopathy and LVH, CKD or stroke. CKD was the most significant predictor of hypertensive retinopathy and there was a tendency toward increased risk of target-organ damage among patients with advanced hypertensive retinopathy.

## Abstract

Hypertension is a major public health problem worldwide and on the African continent.^1^,^2^ The disease, once considered to be rare outside Europe and North America, is now a leading cause of disability and mortality in developing countries. Its prevalence is projected to reach 30% worldwide by 2025.^2^

Poor control of hypertension increases the likelihood of complications affecting the cardiovascular and cerebrovascular systems, kidney and retina, often labelled under the term target-organ damage (TOD).^1^ The development of subclinical TOD, such as left ventricular hypertrophy (LVH), increased intima–media thickness of the large vessels, microalbuminuria following glomerular dysfunction, cognitive decline and hypertensive retinopathy precedes the occurrence of major complications, which include stroke, congestive heart failure and myocardial infarction, renal failure and retinal vascular occlusions.^3^-^5^ In the Democratic Republic of Congo (DRC), the prevalence of systemic hypertension has been reported to be over 25%,^6^,^7^ whereas hypertension and associated complications account for over 20% of deaths among adults.^8^

Studies have demonstrated that TOD increases cardiovascular risks over that already associated with elevated blood pressure alone. For example, it has been shown that once LVH has developed following long-standing systemic hypertension, it behaves as an independent risk factor and a predictor of both further cardiac complications,^9^ and other incident vascular events such as ischemic stroke and myocardial infarction.^10^ Similarly, the presence of cerebrovascular and renal damage may raise cardiovascular risk over that conferred by hypertension itself.^11^,^12^

In addition, hypertensive retinopathy has long been known as a predictor of systemic morbidity and mortality. Both epidemiological and clinical studies have provided evidence that markers of hypertensive retinopathy are associated with raised blood pressure, systemic vascular diseases, and subclinical cerebrovascular and cardiovascular disease, and predict incident clinical stroke, congestive heart failure and mortality due to cardiovascular complications.^13^ This association of hypertensive retinopathy with other TOD has also been shown to be independent of blood pressure and other risk factors, which supports the recommendation that retinal vascular changes should be assessed in individuals with systemic hypertension for better extra-ocular TOD risk stratification.^13^

While the number of reports on hypertensive TOD has been on the rise on the African continent, the relationship between hypertensive retinopathy and other TOD has largely remained unexplored. The aim of this study was to examine the association of hypertensive retinopathy with LVH, chronic kidney disease (CKD) and stroke in Congolese patients.

## Methods

This cross-sectional, observational study included 159 consecutive Congolese hypertensive patients (73 men, 86 women, mean age 57.9 ± 13.2 years) who were referred from the Cardiology Division to the Ophthalmology Department of the Kinshasa University Hospital for fundus examination as part of a work-up of people with hypertension. All participants provided informed consent and the study was approved by the University of Kinshasa Medical School institutional review board.

Inclusion criteria were age ≥ 18 years, willingness to participate in the study, established diagnosis of hypertension regardless of treatment regimen, duration, severity or aetiology. Exclusion criteria included inaccessibility of the fundus due to media opacities, and pregnancy.

All participants underwent blood pressure measurement with a mercury sphygmomanometer after the patient has been in a sitting position for five minutes, and body mass index (BMI) determination. They provided personal information about history of alcoholism, smoking, as well as family history of hypertension and stroke, and diabetes.

Routine ophthalmological examination was performed, which included measurement of visual acuity, slit-lamp examination of the anterior segment, intra-ocular pressure measurement with applanation tonometry, and ocular fundus assessment with direct ophthalmoscopy after pupil dilation with tropicamide 1% and phenylephrine 10%. The fundus examination specifically looked at retinal abnormalities consistent with hypertensive retinopathy, which was graded based on the Scheie classification:^14^ grade 0 = no visible change; grade 1 = barely detectable arterial narrowing; grade 2 = obvious arterial narrowing with focal irregularities; grade 3 = grade 2 plus retinal haemorrhages, exudates, cotton wool spots, or retinal oedema; grade 4: grade 3 plus papilloedema.

Hypertension was defined and classified according to the European Society of Hypertension/European Society of Cardiology guidelines.^15^ Data about extra-ocular TOD such as LVH, CKD and stroke were recorded from cardiology medical records. LVH was diagnosed by echocardiogram using ASE criteria:^16^ end-systolic left ventricular diameter, septal wall thickness (SWT) and posterior wall thickness were calculated from the two-dimensionnally guided M-mode tracing and measured in five consecutive cardiac cycles. LVH was defined by SWT greater than 11 mm.

CKD was diagnosed according to the Kidney Disease Outcome Quality Initiative (K/DOQI),^17^ when glomerular filtration rate (GFR) was lower than 60 ml/min/1.7 m^2^ using the equation from the Modified Diet in Renal Disease (MDRD) study.^18^ Stroke was diagnosed in the presence of clinical neurological signs consistent with stroke, with or without supporting CT scan lesions.

## Statistical analysis

All analyses were performed with SPSS version 15.0 (SPSS, Chicago, IL, USA). Data were expressed as mean ± standard deviation. Student’s t-test was used to compare means between groups. The proportion of patients with hypertensive retinopathy was compared among those with and without LVH, CKD or stroke using the chi-square test. The chi-square test was also used to compare the proportions of patients with TOD between those with and without hypertensive retinopathy. Multiple logistic regression analysis allowed assessment of the association of demographic and clinical factors including TOD with the likelihood of having hypertensive retinopathy. A *p* < 0.05 was considered statistically significant.

## Results

Of the 159 patients included in this study, 73 (46%) were male and 86 (54%) were female, with a mean age of 57.9 ± 13.2 years (range: 19–92). Approximately half of the patients (48.4%) had been hypertensive for one to 10 years; 137 (86.2%) patients had essential hypertension and 22 were diabetic. Hypertension was grade 1 (systolic: 140–159 mmHg and diastolic: 90–99 mmHg), grade 2 (systolic: 160–179 mmHg, diastolic: 100–109 mmHg) and grade 3 (systolic ≥ 180 mmHg, diastolic ≥ 110 mmHg) in 48 (30.2%), 34 (21.4%) and 77 (48.4%) patients, respectively. One hundred and twenty-two (76.7%) patients were on blood pressure-lowering treatment (57.2% had uncontrolled whereas 19.5% has controlled blood pressure) and 37 (23.3%) were not on treatment at the time of study enrolment.

Other characteristics of the study population were as follows: weight 71.8 ± 16.3 kg (range: 42–130), height 163.5 ± 8.9 cm (range: 148–162), waist circumference 90.9 ± 12.8 cm (range: 67–125), systolic blood pressure 159.1 ± 30.9 mmHg (range: 100–230), diastolic blood pressure 95.1 ± 16.6 mmHg (range: 61–157), serum creatinine 2.2 ± 3.6 mg/dl (range: 0.3–19) and blood urea 38.9 ± 42.1 mmol/l (range: 5.2–258).

Hypertensive retinopathy stage 0, 1, 2, 3 and 4 was present in 16.4, 42.1, 11.3, 23.3 and 6.9% of the patients, respectively. Overall, the severity of hypertensive retinopathy increased with increasing systolic and diastolic blood pressures. Data on cardiac state were available for 97 (61%) patients, of whom 52 (53.6%) had LVH. Twenty-eight (31.8%) of the 88 patients who underwent glomerular filtration rate (GFR) assessment had levels consistent with CKD and 28 (17.6%) patients were diagnosed as having stroke.

Table 1 shows the distribution of patients with LVH by stage of retinopathy. The proportions of patients with retinopathy were comparable among those with (86.5%) and without LVH (73.3%) (χ^2^ = 1.53, *p* = 0.21). Similarly, the proportions of patients with LVH did not differ significantly between patients with (57.7%) and those without retinopathy (36.8%) (χ^2^ = 0.39, *p* = 0.53). There was no significant association between hypertensive retinopathy and LVH (χ^2^ = 1.9, *p* = 0.17, OR = 2.3, 95% CI: 0.8–6.6). For all retinopathy stages, the proportions of patients with and without LVH were comparable (*p* = 0.24–0.99, data not provided in Table 1). The risk of having LVH tended to increase with the severity of hypertensive retinopathy; it was 4.5 times higher for patients with grade 3 hypertensive retinopathy relative to those without retinopathy.

**Table 1:** Association between hypertensive retinopathy and left ventricular hypertrophy (LVH )

*Retinopathy grade*	*With LVH (%)*	*Without LVH (%)*	*OR (95% CI)*	*Chi-square*	p*-value*
0	7 (13.5)	12 (26.7)	1	-	-
1	19 (36.5)	21 (46.7)	1.6 (0.5–4.8)	0.24	0.62
2	10 (19.2)	5 (11.1)	3.4 (0.8–14.2)	1.91	0.17
3	13 (25.0)	5 (11.1)	4.5 (1.1–17.9)	3.34	0.07
4	3 (5.8)	2 (4.4)	2.6 (0.3–19.3)	0.18	0.67

OR: odd ratio, CI: confidence interval.

There were 28 patients with CKD; their distribution by retinopathy stage is provided in Table 2. Subgroups of patients with and without retinopathy had similar proportions of patients with CKD (33.3 vs 23.1%) (χ^2^ = 0.088, *p* = 0.77). A similar observation was made regarding the proportions of patients with hypertensive retinopathy among those with and without CKD (89.3 vs 83.3%) (χ^2^ = 0.12, *p* = 0.73). The association of CKD with hypertensive retinopathy was not significant for all retinopathy stages combined (χ^2^ = 0.17, *p* = 0.68, OR = 1.7, 95% CI: 0.4–6.6) or for each retinopathy stage taken individually (χ^2^ = 0.03–2.82, *p* = 0.09–0.85). Compared to patients without hypertensive retinopathy, those with stages 3 and 4 hypertensive retinopathy were 3.3 and 13.3 times more likely to have CKD, respectively.

**Table 2:** Association between hypertensive retinopathy and chronic kidney disease (CKD)

*Retinopathy grade*	*With CKD (%)*	*Without CKD (%)*	*OR (95% CI)*	*Chi-square*	p*-value*
0	3 (10.7)	10 (16.6)	1	–	–
1	9 (32.1)	26 (43.3)	1.2 (0.3–5.2)	0.04	0.85
2	2 (7.1)	13 (21.7)	0.5 (0.07–3.7)	0.03	0.86
3	10 (35.7)	10 (16.7)	3.3 (0.7–15.9)	1.4	0.24
4	4 (14.3)	1 (1.7)	13.3 (1.1–169.1)	2.8	0.09

OR: odd ratio, CI: confidence interval.

There were 85.7% of patients with hypertensive retinopathy among those who suffered from stroke (28 patients, Table 3). This proportion was not significantly different from the 83.2% of patients with hypertensive retinopathy among those without stroke (χ^2^ > 0.001, *p* = 0.99). Patients with hypertensive retinopathy were as likely as those without retinopathy to have stroke (18 vs 15.4%) (χ^2^ = 0.34, *p* = 0.56). No association was found between stroke and hypertensive retinopathy regardless of retinopathy stage (χ^2^ < 0.01, *p* = 0.96, OR = 1.2; 95% CI: 0.4–3.8) and for individual retinopathy stages (χ^2^ = 0.02–1.06, *p* = 0.30–0.88).

**Table 3:** Association between hypertensive retinopathy and stroke

*Retinopathy grade*	*With LVH (%)*	*Without LVH (%)*	*OR (95% CI)*	*Chi-square*	p*-value*
0	4 (14.3)	22 (16.8)	1	-	-
1	11 (39.3)	56 (42.7)	1.1 (0.3–3.8)	0.04	0.85
2	6 (21.4)	12 (9.2)	2.8 (0.7–11.7)	1.1	0.30
3	4 (14.3)	33 (25.2)	0.7 (0.2–2.9)	0.02	0.88
4	3 (10.7)	8 (6.1)	2.1 (0.4–11.3)	0.2	0.70

OR: odd ratio, CI: confidence interval.

A subset of data of 75 patients with complete documentation was used to perform a multivariate logistic regression analysis that included age, gender, BMI, alcohol consumption, smoking, diabetes, arterial pressures (systolic, diastolic and pulse), current blood pressure-lowering treatment, LVH, CKD and stroke as candidate explanatory variables, and hypertensive retinopathy as outcome variable after controlling for diabetes. The results, shown in Table 4, indicate that CKD was the most significant predictor of hypertensive retinopathy, with OR of 4.4 compared to CKD-free patients. Age > 50 years and smoking appeared to decrease the risk of hypertensive retinopathy; the effects were negligible but significant.

**Table 4:** Significant determinants of hypertensive retinopathy

*Parameters*	*β*	*p-value*	*OR (95% CI)*
Constant	–0.88	0.23	0.41
Chronic kidney disease	1.49	0.018	4.4 (1.29–15.21)
Age > 50 years	–1.46	0.046	0.23 (0.06–0.97)
Smoking	–2.02	0.035	0.1 (0.02–0.9)

OR: odd ratio, CI: confidence interval.

## Discussion

Hypertension is an important cause of morbidity and mortality in the general population in Western countries, and recent surveys in sub-Saharan Africa have reported high prevalences of hypertension ranging between 19 and 50% in both urban and rural populations.^19^,^20^ If left untreated, hypertension may result in considerable damage to the cardiovascular, renal and cerebrovascular systems, leading to such complications as myocardial infarction, CKD and cerebrovascular accident.

While significant efforts have been invested to demonstrate the benefits of antihypertensive treatment, it is critical for better management to know both to what extent the various hypertension-related TODs are interrelated, and the risk factors for hypertension-related damage. Because studies in this regard are limited in sub-Saharan Africa, we investigated the relationship between hypertensive retinopathy and LVH, CKD and stroke among Congolese patients. We also assessed the determinants of hypertensive retinopathy.

It has been hypothesised that both hypertension-related retinal and renal vascular changes share common pathogenetic mechanisms. As a result, earlier studies have consistently reported an association between the presence of retinal vascular changes associated with hypertension and lower GFR.^21^-^23^ Surprisingly, our results suggest otherwise, which may be ascribed to the small study population.

Signs of hypertensive retinopathy have also long been recognised as risk indicators of LVH, both in population- and hospital-based studies.^24^-^26^ For example, in the Chronic Renal Insufficiency Cohort (CRIC) study,^26^ there was an association between severity of hypertensive retinopathy and the incidence of any cardiovascular disease. Similarly, a follow up of the National Health and Nutrition Survey (NHANES I) reported an increased risk of cardiovascular disease in people with hypertensive ocular fundus retinal vascular changes.^27^

The lack of association between hypertensive retinopathy and LVH found in our study echoes the findings of other earlier studies.^28^,^29^ While there is a general agreement on the association between hypertensive retinopathy and all types of hypertensive cardiovascular diseases,^26^,^27^,^30^ our study only focused on LVH, which may explain the lack of association. Overall, our findings corroborate those of earlier studies that the risk of developing LVH increases significantly with the severity of hypertensive retinopathy.

In support of Cuspidi *et al.*,^31^ the frequency of severe retinopathy (i.e. grade 4) appeared to be low among subjects with LVH. The same observation was made among subjects with CKD as well as those with stroke. While we do not have a definitive explanation for this observation, it is possible that hypertensive patients in our setting have a reduced life expectancy so that severe retinopathy has no time to develop.

Hypertensive retinopathy had no association with stroke in this study, which is at odds with reports from earlier investigations. Indeed, many cross-sectional studies have demonstrated a clear relationship between hypertensive ocular fundoscopic abnormalities and both clinical and subclinical stroke, even after adjusting for other independent vascular risk factors.^32^-^34^ However, definitive convincing evidence in favour of this association has been provided by longitudinal studies.^35^-^39^

Unlike most of these earlier studies that used ocular fundus photography and brain imaging techniques to increase the diagnostic accuracy, our diagnosis of stroke was clinical and retrospective in nature. As a result, a substantial number of patients who suffered subclinical stroke and/or hypertensive retinopathy, identifiable using imaging techniques, may have been unaccounted for. The cost of medical imaging modalities such as CT scans limits the patient’s access to this sensitive diagnostic tool. This limitation is also valid for the association between LVH and hypertensive retinopathy.

Studies on predictors of hypertensive retinopathy have reported conflicting results. For example, while aging, obesity measured by BMI, and smoking have been traditionally associated with increased risk of hypertensive retinopathy, Sharp *et al.*^40^ found that age and systolic blood pressure did not influence hypertensive retinopathy in people of African origin, despite a higher prevalence of hypertensive retinopathy in this group compared to people of European descent.

In the ARIC study,^35^ only mean blood pressure was associated with hypertensive retinopathy in the subset of participants of African descent. LVH and BMI were not significant determinants, and smoking had a marginally non-significant effect. The risk-reducing effect of aging, smoking, and LVH on retinopathy that we found is surprising and adds to existing inconsistencies in results across studies. We speculated that higher mortality rates, selectively affecting older people as a result of hypertension-related complications, and other morbidities in our setting may contribute to the inverse ORs observed for age and LVH.

Because arteriolar narrowing and arteriovenous nipping can be found in the absence of hypertension, it has been argued that these signs have little or no value in the management of hypertension, and that clear evidence is lacking to show that patients with mild hypertensive retinopathy need physician referral or follow up. Conversely, landmark prospective studies have provided evidence of the clinical value of retinal arteriolar narrowing. For example, in the Beaver Dam Eye study,^41^ the fiveyear incidence of retinopathy in general and that of arteriolar narrowing was significantly higher in patients with elevated blood pressure, despite being on antihypertensive treatment, relative to those with controlled blood pressure and those with no hypertension.

The Blue Mountain Eye study^42^ reported an association between generalised retinal arteriolar narrowing at baseline and about a three-fold increased risk of five-year incidents of severe hypertension. These findings emphasise the clinical value of assessing retinal arteriolar change for cardiovascular risk prediction, and are supported by international guidelines for hypertension management such as the US Joint National Committee on Prevention, Detection, Evaluation and Treatment of High Blood Pressure, the European Society of Cardiology, the European Society of Hypertension, and the British Society of Hypertension.

We acknowledge that this study has some limitations. The diagnosis of hypertensive retinopathy, particularly in the early stages, has been shown to suffer from high rates of inter- and intra-observer variability when assessed with direct ophthalmoscopy, as in this study. Because only one observer made the assessment and there was no intra-observer, the results presented herein did not account for the possible effect of low reliability. An additional limitation that may have influenced the results is the small number of study participants who underwent GFR assessment and echocardiogram, which may limit the generalisability of our findings.

## Conclusion

There was no association between hypertensive retinopathy and LVH, CKD or stroke in this series. There was a trend towards increased risk for developing TOD among people with advanced retinopathy. CKD emerged as the only significant predictor of hypertensive retinopathy.
